# Comparative proteomics analysis of dietary restriction in *Drosophila*

**DOI:** 10.1371/journal.pone.0240596

**Published:** 2020-10-16

**Authors:** Yue Gao, Chenxing Zhu, Keqin Li, Xingyi Cheng, Yanjiao Du, Deying Yang, Xiaolan Fan, Uma Gaur, Mingyao Yang

**Affiliations:** 1 Institute of Animal Genetics and Breeding, Sichuan Agricultural University, Chengdu, China; 2 Farm Animal Genetic Resources Exploration and Innovation Key Laboratory of Sichuan Province, Sichuan Agricultural University, Chengdu, China; Inha University, REPUBLIC OF KOREA

## Abstract

To explore the underlying mechanism of dietary restriction (DR) induced lifespan extension in fruit flies at protein level, we performed proteome sequencing in *Drosophila* at day 7 (young) and day 42 (old) under DR and *ad libitum* (AL) conditions. A total of 18629 unique peptides were identified in Uniprot, corresponding to 3,662 proteins. Among them, 383 and 409 differentially expressed proteins (DEPs) were identified from comparison between DR vs AL at day 7 and 42, respectively. Bioinformatics analysis revealed that membrane-related processes, post-transcriptional processes, spliceosome and reproduction related processes, were highlighted significantly. In addition, expression of proteins involved in pathways such as spliceosomes, oxidative phosphorylation, lysosomes, ubiquitination, and riboflavin metabolism was relatively higher during DR. A relatively large number of DEPs were found to participate in longevity and age-related disease pathways. We identified 20 proteins that were consistently regulated during DR and some of which are known to be involved in ageing, such as mTORC1, antioxidant, DNA damage repair and autophagy. In the integration analysis, we found 15 genes that were stably regulated by DR at both transcriptional as well as translational levels. Our results provided a useful dataset for further investigations on the mechanism of DR and aging.

## 1. Introduction

Dietary Restriction (DR) is broadly defined as reducing 20%-40% of food intake without causing malnutrition. In 1930s, it was first demonstrated that DR prolonged lifespan and improved the age-related phenotype in mice [1]. In recent years, DR has been proved to be effective in delaying aging and extending lifespan in a variety of organisms such as nematodes [2], fruit flies [3], fish [4], dogs [5], rodents [1] and primates [6]. Interestingly, many age-associated diseases such as arteriosclerosis [7], neurodegenerative diseases [8], liver deformities [9] and type 2 diabetes [10] have shown improvement under DR. In addition, DR can also prevent and delay some cancers [11]. Even though it has been known previously that DR affects multiple evolutionarily conserved signaling pathways yet [12–15], the currently available studies could not fully explain the changes brought by dietary restriction, and many mechanisms remain unclear.

Although our previous studies have reported and explained the changes induced by DR at whole-genome transcriptome levels [3], it is believed that proteins are directly involved in biological processes. Therefore, the proteome of an organism can offer more insight into the life activities of the organism than the genome and the transcriptome. Recently, the omics studies have confirmed that DR exerts a variety of benefits including anti-aging. The metabolomics analysis of rat urine, blood and liver reveals some of the benefits of DR in liver detoxification [16]. Region-specific proteome changes in mice intestinal epithelium reveal the effect of DR on the partial restoration of epithelium during aging [17]. It was also reported that the decline of protein homeostasis and mitochondria are critical signals prior to onset of aging and aging-related diseases through multiple proteomics analysis on fly [18]. Proteome analysis have been carried out in fruit-flies in the context of age-related diseases, such as Alzheimer’s disease [19]. By measuring the head proteome of adult fly, Brown et al [20] revealed that protein homeostasis decreases with age in fly head. However, there is a lack of studies that combine DR with aging to determine proteome changes in the overall body in fly.

In this study, the isobaric tags for relative and absolute quantification (iTRAQ) technology was chosen to sequence the proteomes [21], which can utilize isobaric reagents to label the primary amines of peptides and proteins. Its advantage is that it can analyze 8 samples simultaneously using 8-plex iTRAQ reagent [21,22]. We used iTRAQ method to sequence the proteomes of *Drosophila melanogaster* under DR and *ad libitum* (AL) conditions at different life stages. As mentioned in our pervious report [3], flies at the day 7 have been considered as young flies and the flies in the present study began to show aging associated changes at the day 42. Thus, we choose same time points of day 7 and day 42 for carrying out aging specific analysis involving expression patterns of proteomics dynamics. The proteome of *Drosophila* was compared at the day 7 and 42 on DR and AL conditions, respectively. A relatively large number of differentially expressed proteins (DEPs) were found to participate in longevity pathway and age-related disease pathways. The results from the present study can offer further insight into how flies respond to DR in the young and old life stages and DR associated benefits in maintaining health and delaying the process of aging.

## 2. Materials and methods

### 2.1 Fly management and foods

Wild-type Dahomey *Drosophila melanogaster* stock maintenance and handling procedures were described in Bass et al [23]. In short, female flies used in experiments were cultured at 25°C on a constant light: dark cycle of 12:12h at 65% humidity. In experiments, dietary restriction medium (DR, 1xSYA) contained 100g/l yeast (1x; MP Biomedicals, USA), 50 g/l sucrose (Sigma-Aldrich, USA), 1.5% agar, (BioFroxx, Germany), 30ml/l nipagin (Micxy Chemical, Chengdu, China) and 3ml/l propionic acid (Chron Chemicals, Chengdu, China). The *ad libitum* medium (AL, 2xSYA) was prepared in the same way, except that it contained 200g/l yeast. 1xSYA food were also used for general culture for flies.

### 2.2 Lifespan assay

The experimental procedure refers to the method of Emran, et al [13]. Briefly, lifespan assay was performed under DR and AL conditions. For each condition, 100 flies were used. Log-rank test was used for comparison of survivorship data.

### 2.3 Sample collection

*Drosophila* samples under DR and AL conditions were collected at day 7 and day 42. Each sample was prepared by pooling 10 female fruit flies. A total of 8 samples (four samples in two replicates) were included. They were named as d7 AL1^st^, d7 AL 2^nd^, d7 DR 1^st^, d7 DR 2^nd^, d42 AL1^st^, d42 AL 2^nd^, d42 DR 1^st^, d42 DR 2^nd^, respectively. The protein samples of whole fruit fly were extracted for subsequent experiments.

### 2.4 Protein extraction

Each sample was homogenized in 200μl of TBS buffer on ice. After centrifugation at 1000 rpm at 4°C for 10 minutes, 200μl of SDT-lysis buffer (30g/l SDS, 0.1mol/l Tris-HCl pH = 7.6, 0.1mol/l DTT) was added and the sample was heated at 80°C for 5min. After cooling down the sample, 20μl of 50mM IAA was added and the clear lysate was obtained by centrifugation at maximum speed for 5 minutes. The clarified lysate was taken out and incubated in the dark for 30min. The protein concentration of each samples was measured using a nucleic acid protein analyzer. Protein concentrations obtained were high enough for the subsequent processes (S1 Table).

### 2.5 Sample digestion and extraction

Standard operating procedure for SDS-PAGE (Roche Life Science) was followed for detecting the proteins.

Dried gels were completely swollen on ice using 12.5μg/μl of trypsin solution (50mM NH4HCO3, 5% acetonitrile, 10 ng/μl trypsin). Then 10μl of 50mM NH4HCO3 was added to ensure that the pH was slightly above 7. Proteins were fully digested for about 12 hours at 37°C. The supernatant containing peptides was obtained by centrifugation at 13000rpm for 1min. 40μl of extraction solution (5ml of formic acid, 50ml of acetonitrile and 45ml of ultrapure water in total 100ml) was added to the remaining gels at the bottom of the EP tube for further extraction and kept for 5 min. The supernatant was obtained by centrifugation at 13000rpm for 1min. The peptide was repeatedly extracted until the gels became dry and hard. The extracted peptide solution was concentrated and dried into dry powder. The final sample was stored at -80°C for iTRAQ experiment.

### 2.6 Mass spectrometer data acquisition

For iTRAQ labeling, 100μg peptides of each sample were labeled with iTRAQ Reagents (Applied Biosystems). Eight groups of iTRAQ labeled peptides were mixed in equal proportions by vortex. The peptide mixture was freeze-dried in vacuum.

iTRAQ labeled peptides were fractionated by Strong Cation Exchange (SCX) chromatography using the AKTA Purifier system (GE Healthcare). Briefly, dried peptide mixture was dissolved completely with Buffer A (10 mM KH2PO4 in 25% of ACN, pH 3.0) and loaded onto a PolySULFOETHYL 4.6 x 100 mm column (5μm, 200 A, PolyLC Inc, Maryland, U.S.A.). The flow rate was 1mL/min. The liquid phase gradients were 0%–8% buffer B (500 mM KCl, 10 mM KH2PO4 in 25% of ACN, pH 3.0) for 22 min, 8–52% buffer B during 22–47 min, 52%–100% buffer B during 47–50 min, 100% buffer B during 50–58 min, and buffer B was reset to 0% after 58 min. The absorbance of the eluent was detected at 214nm. Fractions were collected every 1 min. Then the collected fractions were combined into 20 parts, which were desalted using C18 Cartridge (Empore™ SPE Cartridges C18 (standard density), bed I.D.7 mm, volume 3ml, Sigma).

20 fractions were separately analyzed by nanoLC-MS/MS. Briefly, the peptide mixture was loaded onto a reverse phase trap column (Thermo Scientific Acclaim PepMap100, 100μm*2cm, nanoViper C18) connected to the C18-reversed phase analytical column (Thermo Scientific Easy Column, 10 cm long, 75μm inner diameter, 3μm resin) in buffer A (0.1% Formic acid) and separated at a flow rate of 300nl/min with a linear gradient of 0%–35% buffer B (84% acetonitrile and 0.1% Formic acid) for 50 min, 35–100% buffer B during 50–55 min, 100% buffer B during 55–60 min.

The samples after chromatographic separation are analyzed by Q-Exactive mass spectrometer (Thermo Scientific) for 1h. The MS was set in positive ion mode. MS data was acquired using a data-dependent top10 method dynamically choosing the most abundant precursor ions from the survey scan (300–1800 m/z) for HCD fragmentation. Automatic gain control (AGC) target was 3e6. Maximum inject time (IT) was 10 ms. Number of scan ranges was 1, Dynamic exclusion duration was 40.0s. Survey scans were acquired at a resolution of 70,000 at m/z 200 and resolution for HCD spectra was set to 17,500 at m/z 200. Isolation window was 2m/z. Normalized collision energy was 30eV and the underfill ratio was 0.1%. The instrument was run with peptide recognition mode enabled. The mass spectrometry proteomics data has been deposited to the ProteomeXchange Consortium via the PRIDE [24] partner repository with the dataset identifier PXD021022.

### 2.7 Mass spectrometer data analysis

Mass spectra results among two biologically repeated samples were consistent. Qualitative analysis of protein was carried out using Mascot 2.2 identification parameters. The original spectrum of mass spectrum was searched in Uniprot database for *Drosophila melanogaster* using Proteome Discoverer 1.4 (Thermo Scientific). To obtain reliable qualitative results, the screening standard was set at FDR<0.01. For protein quantitative analysis, Proteome Discoverer 1.4 software was used to extract the peak intensity value of peptide reporter ion. The quantitative value of day 7 DR, day 42 AL and day 42 DR of each repeat was normalized to the average value of day 7 AL.

### 2.8 Bioinformatics analysis

Protein quantification of each comparison group was taken as the average value of two biological replicates. We conducted Significance A analysis of the DEPs in 4 comparison groups (day 7 DR vs AL, day 42 DR vs AL, day 42 vs day 7 AL, and day 42 vs day 7 DR), using ratio >1.2 and <0.8 (*p*<0.05) as cutoffs. Annotation and pathway enrichment of DEPs was performed using Gene Ontology (GO) and the KEGG DAVID tool. The significance of enrichment was obtained by Fisher's exact test (*p*<0.05). Cluster analysis was performed on DEPs. Transcriptome data set obtained from our previously report at the same time point of d7 and d42 under DR condition [3].

## 3. Result

### 3.1 Dietary restriction extends fly lifespan

**Fig 1:**
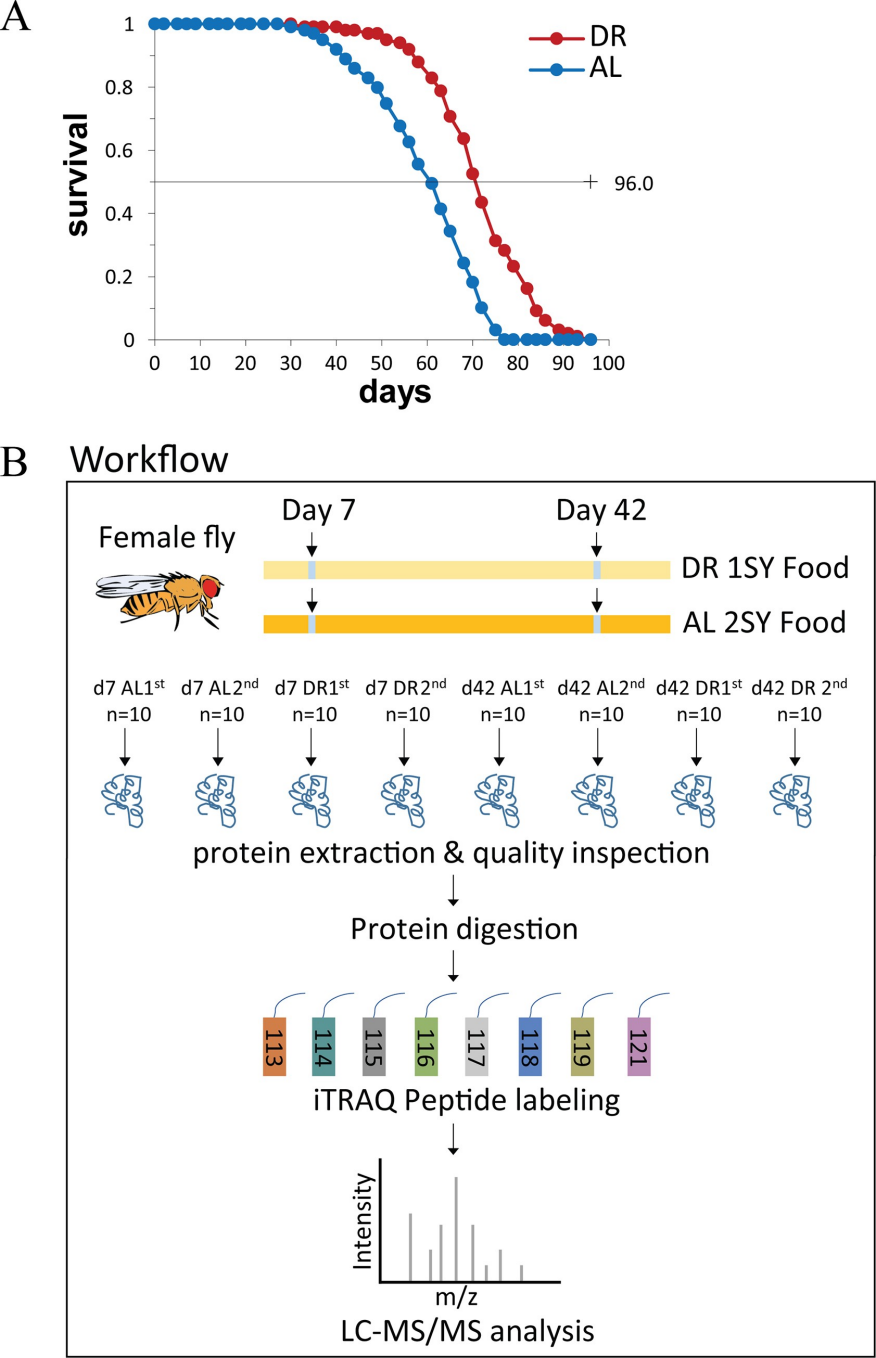
Lifespan curve and workflow of proteome sequence. **(**A) DR extends the lifespan in fruit flies. Curve of lifespan, DR (red, 1SYA food), AL (blue, 2SYA food). n = 100, p≤0.01. (B) Workflow from sample collection to mass spectrometry data acquisition. Details can be found in methods.

Dietary restriction extended *Drosophila* median lifespan by 19.3% in comparison to fully fed flies in the present study (Fig 1A, S2 Table), which is similar to the previous report [3]. In order to systematically understand the changes of *Drosophila* response to DR at the protein level, we carried out the proteome sequencing and compared the DEPs in fruit fly under DR and AL conditions at day 7 and day 42 respectively (Fig 1B).

### 3.2 Differentially expressed proteins analysis

By comparing *Drosophila melanogaster* data in Uniprot database, a total of 22010 peptides were identified (S3 Table), of which 18629 were unique peptides (S4 Table). Finally, 3662 proteins were identified, of which 99.6% of the proteins were identified in each sample, indicating that the sample consistency is reliable (S4 Table).

**Fig 2:**
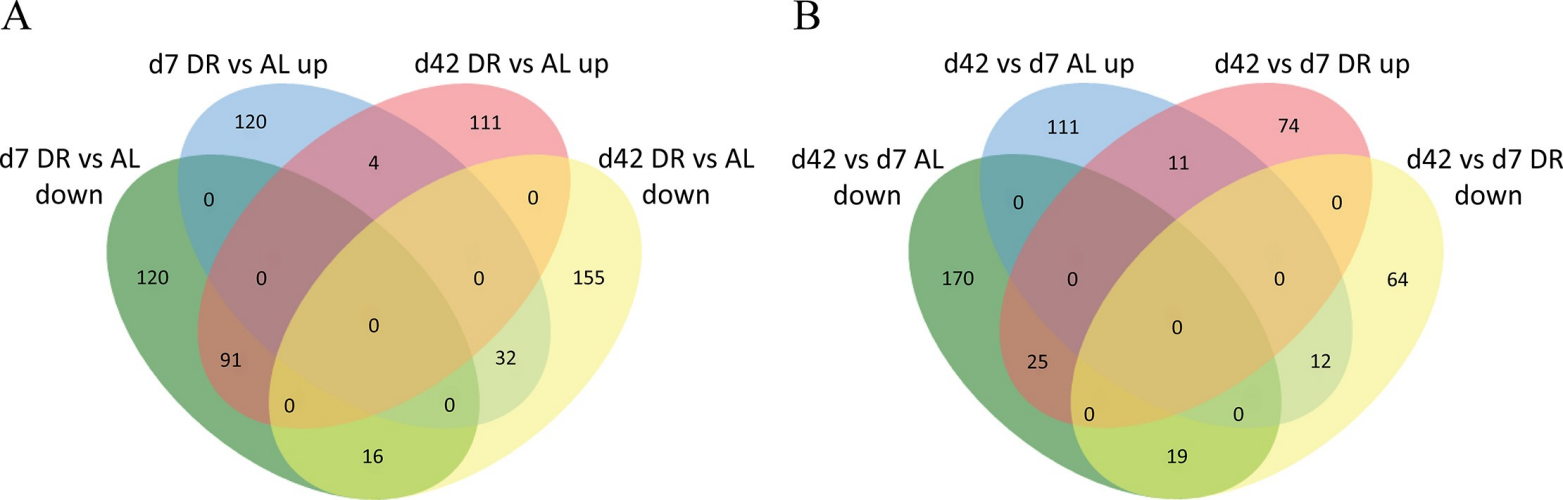
Venn diagram of DEPs. (A) Diet comparison between DR vs AL at day 7 and DR vs AL at day 42, (B) Age comparison, DR between d42 vs d7 and AL between d42 vs d7.

The comparison analysis included 4 groups d7 DR vs AL, d42 DR vs AL, d42 vs d7 AL, and d42 vs d7 DR (S5 Table). Using ratio >1.2 and <0.8 (p<0.05) as cutoffs, in diet comparison, 383 DEPs were identified in d7 DR vs AL, of which 156 were up-regulated and 227 were down- regulated. A total of 409 DEPs were observed in the d42 DR vs AL comparison, of which 206 were up-regulated and 203 were down-regulated (Table 1, S1A and S1B Fig). In terms of age comparison, a total of 205 DEPs, of which 110 were up-regulated and 95 were down-regulated, were identified at d42 vs d7 within DR. A total of 348 DEPs, of which 134 were up-regulated and 214 were down-regulated were found at d42 vs d7 within AL (Table 1, S1C and S1D Fig). Analysis with Venn diagram showed that d7 DR vs AL and d42 DR vs AL shared 4 common up-regulated and 16 common down-regulated DEPs, indicating that these DEPs might play general regulatory roles during DR (Fig 2A). Also, 11 up- and 19 down-regulated DEPs were shared by d42 vs d7 AL and d42 vs d7 DR, suggesting that their expression might be only related to age. In contrast, 74 up- and 64 down- regulated DEPs only appeared in d42 vs d7 DR comparison, indicating that these proteins are regulated by DR (Fig 2B).

**Table 1:** Protein quantitative results.

	up	down	total
d7 DR vs AL	156	227	383
d42 DR vs AL	206	203	409
DR d42 vs d7	110	95	205
AL d42 vs d7	134	214	348

### 3.3 GO annotations of DEPs between DR and AL

**Fig 3:**
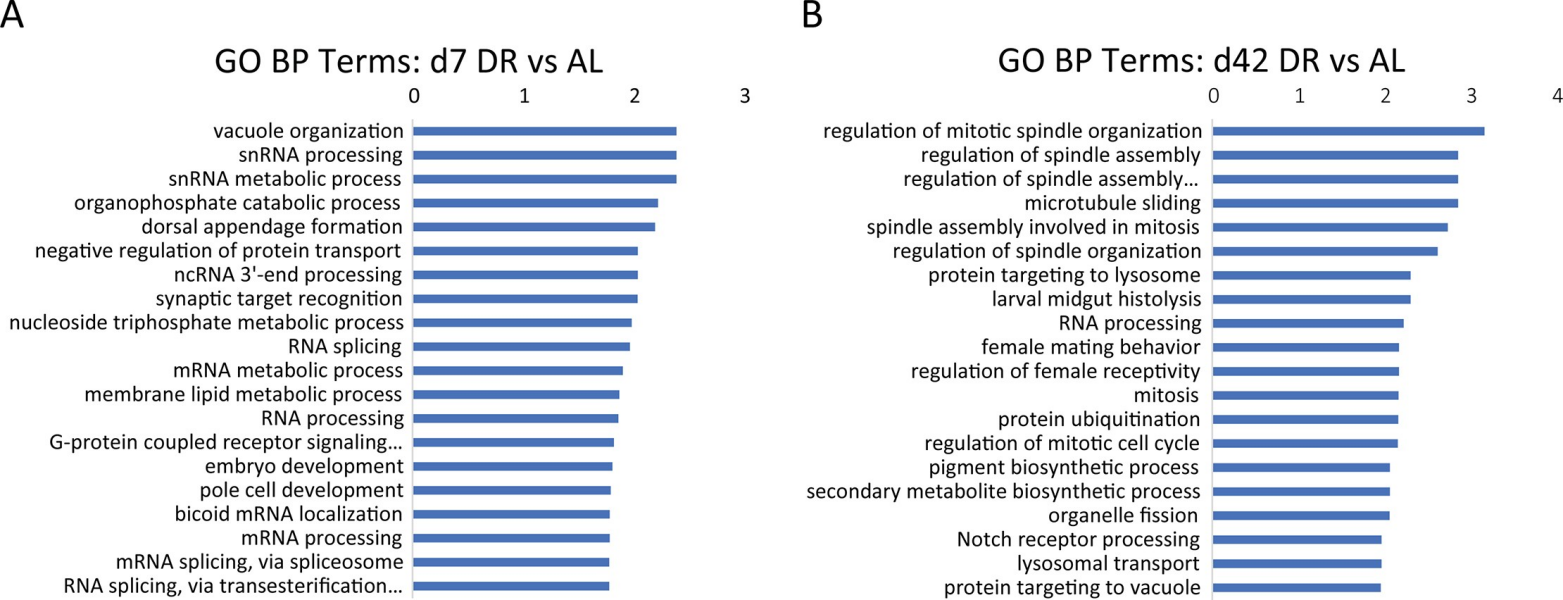
Top 20 significantly enriched GO biological processes. (A) d7 DR vs AL and (B) d42 DR vs AL (p<0.05). Results are presented as -Log10 (P value). The full names of the abbreviated processes with an ellipsis are “G-protein coupled receptor signaling pathway, RNA splicing”, “via transesterification reactions with bulged adenosine as nucleophile”, and “regulation of spindle assembly involved in mitosis”.

To determine the key processes involved in DR, we performed GO enrichment on 383 DEPs at d7 DR vs AL and 409 DEPs at d42 DR vs AL. We then assessed the top 20 significantly enriched Gene Ontology Biological Process (GO BP) of d7 DR vs AL (Fig 3A, S6 Table) and d42 DR vs AL (Fig 3B, S6 Table). The top enriched process were vacuole organization and snRNA associated process in d7 DR vs AL. The next were membrane lipid metabolic process, and post-transcriptional related processes (ncRNA 3'-end processing, bicoid mRNA localization, several processes related to mRNA processes and RNA splicing). Some important metabolic processes, such as, nucleoside triphosphate metabolic process and organophosphate catabolic process (A0A0B4LHC3 participated in both these processes) were also included. In addition, negative regulation of protein transport, G-protein coupled receptor signaling pathway were also revealed in GO terms.

On the other hand, the most obvious feature was mitosis related processes in d42 DR vs AL. The top 6 enriched process were related to mitotic spindle. Results also showed protein degradation processes, such as, protein ubiquitination, protein targeting to lysosome and lysosomal transport. In line with day 7, d42 was also enriched in a considerable amount of protein participating in the post-transcription process (RNA processing). Enrichment analysis also revealed that DR regulated the reproduction-related processes (regulation of female receptivity, female mating behavior) in fruit flies in old age. In concordance with day 7, membrane-related processes (organelle fission, protein targeting to vacuole) were also enriched. Interestingly enriched notch receptor processing was also included in the GO terms that is crucial for stem cell differentiation.

### 3.4 KEGG pathway analysis of the DEPs between DR and AL

**Fig 4:**
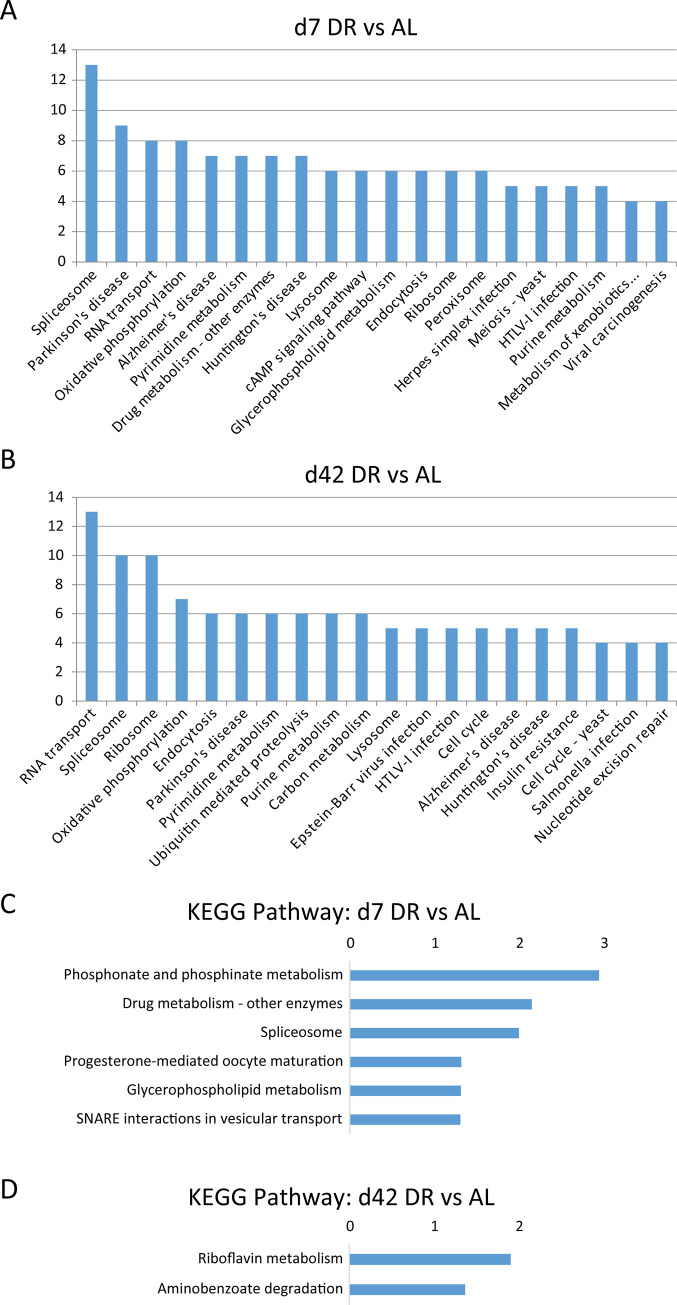
Top 20 KEGG annotations with the most DEPs. (A) d7 DR vs AL. (B) d42 DR vs AL. Results are presented as the number of DEPs. The full names of the abbreviated processes with an ellipsis are “Metabolism of xenobiotics by cytochrome P450”. KEGG pathway enrichment. (C) d7 DR vs AL and (D) d42 DR vs AL, (p<0.05). Results are presented as -Log10 (P value).

In organisms, proteins normally do not act independently, but coordinate with each other in signaling pathways. To understand which signaling pathways are these DEPs involved in, we further conducted KEGG pathway analysis. KEGG analysis showed that DEPs at d7 DR vs AL involved 231 pathways, of which 49 pathways had at least 4 DEPs involved (S6 Table). DEPs at d42 DR vs AL annotated to 209 possible pathways, of which 33 pathways included at least 4 DEPs (S6 Table). According to the top 20 KEGG annotations with the most DEPs (Fig 4A and 4B), at both d7 and d42 DR vs AL not only aggregated on Redox reactions related pathway (Oxidative phosphorylation, Peroxisome) and nucleotide excision repair related pathway (nucleotide excision repair, Pyrimidine metabolism, Purine metabolism), but also enriched on lysosome and viral infection (Herpes simplex infection, HTLV-I infection, Metabolism of xenobiotics by cytochrome P450, Viral carcinogenesis in day 7; Epstein-Barr virus infection, Salmonella infection in d42; Endocytosis of both), and neurodegenerative disease (Parkinson's disease, Alzheimer's disease, Huntington's disease). Glycerophospholipid metabolism pathway and drug metabolism pathway were aggregated in by several DEPs at d7 DR vs AL. Also, the annotation results of d42 DR vs AL group additionally included insulin resistance and ubiquitin mediated proteolysis. It was worth noting that the DEPs of both d7 and d42 DR vs AL were enriched in the spliceosome-related pathways. Previously reported human skeletal muscle proteomic studies also found changes in spliceosomes during aging [25]. Alternatively spliced genes increase with age in mice transcriptome [26]. Since spliceosomes are the main bearers of proteome diversification, in recent years, the biological functions of spliceosomes in aging [27], DR [28], and age-related diseases, such as, cancer [29,30], Alzheimer's Disease [31,32], have been widely studied. Note that some of the spliceosome-related DEPs we screened also showed to be related to aging. For example, O97125 (heat shock protein 68, HSP68) and A0A0B4KFB8 (TCERG1) participated in longevity regulating pathway. Q9VJ12 (ACIN1) was apoptotic chromatin condensation inducer in the nucleus. It will be interesting to explore the role of spliceosome in DR benefits. The DEPs we provided here will serve as crucial DR proteomics profiles for further analysis.

In order to further determine the main biochemical metabolic pathways and signal transduction pathways involved in DEPs, we performed KEGG pathway enrichment on d7 and d42 DR vs AL comparison groups, respectively. Six pathways were found to be enriched in d7 DR vs AL comparison, including phosphonate and phosphinate metabolism, spliceosome, drug metabolism—other enzymes, progesterone-mediated oocyte maturation, glycerophospholipid metabolism, SNARE interactions in vesicular transport (Fig 4C, S6 Table). SNARE has been reported to mediate intracellular membrane fusion [33]. Significantly enriched membrane-related processes, reproduction related processes, and spliceosome are mentioned again. Two pathways which are Riboflavin metabolism and Aminobenzoate degradation, were enriched in d42 DR vs AL (Fig 4D, S6 Table).

### 3.5 DEPs participate in longevity pathway

Many DEPs were involved in longevity-related pathways, including MAPK, insulin/PI3K/Akt, mTOR, FoxO, Wnt and P53 signaling pathways. For example, P92208 (basket, bsk) is a key component of JNK pathway and involves stress response and lifespan determination [34]. The KEGG annotations of P92208 were multiple signaling pathways of insulin, Wnt, mTOR, and Foxo, which involved in the longevity regulation and was significantly down-regulated in the d7 DR vs AL group. Similarly, P12370 (cAMP-dependent protein kinase catalytic subunit 1, Pka-C1) that was significantly up-regulated in the d7 DR vs AL group, was annotated to insulin and Wnt signaling pathway. It was reported to contribute to rhythmic behavior [35] and synaptic related activities. O97125 (HSP68) was seen to be up-regulated in d7 DR vs AL group and is previously suggested to be involved in lifespan determination [34] and response to heat shock and starvation. In d42 DR vs AL group, following genes were significantly up- or down- regulated; C3KKC3 (raptor-RA) which is a crucial component of TORC1 and Q7K2X8 (Nucleoporin at 44A, Nup44A) and is involved in TORC1 signaling and autophagy, significantly up-regulated Q9V998 (Ubiquitin- like protein 5) and down-regulated P48598 (eukaryotic translation initiation factor 4E1, eIF4E1) both participated in the longevity regulating pathway; Q9V8I2 participating in the mTOR signaling pathway was significantly down-regulated in both d7 and d42 DR vs AL comparison group. These DEPs involved in longevity-related pathways may be related to the longevity determination in DR effects and further research evidence is required to validate this hypothesis.

### 3.6 DEPs participate in age-related disease pathways

A large number of DEPs at day 7 between DR vs AL and day 42 between DR vs AL were involved in age-related disease pathways, such as neurodegenerative diseases (Parkinson's disease, Alzheimer's disease, Huntington's disease) and Endocrine and metabolic disease, such as insulin resistance, Non-alcoholic fatty liver disease (NAFLD) and diabetes. In addition, many DEPs at d42 DR vs AL were involved in the cancer pathways. This implied that DR may play a role in resisting or alleviating the disease in the elderly, which is consistent with the previously reported DR benefits to healthy aging [36]. In fact, DR has been reported to provide anti-tumor and beneficial effects in cancer treatment [11,37]. Our proteome sequencing results further provide evidence for the involvement of DR in regulating age-related disease pathways, among which these DEPs may be candidate regulators.

### 3.7 DEPs that were constantly present during DR

To explore the proteins playing a general role during DR, we focused on the proteins mentioned above that changed consistently in response to DR at both d7 and d42. There were four common up-regulated and 16 common down-regulated DEPs. We believe that these DEPs might be basic regulators of important physiological changes caused by DR. We further carried out GO annotations and KEGG pathway annotations of these shared DEPs (Table 2).

**Table 2:** Common DEPs at d7 and d42 between DR vs AL.

UniProt	Protein and Gene Names in Drosophila	KEGG Annotations		Expression level	Significance
		Definition	KEGG pathway		
Q9W138	S-adenosylmethionine sensor upstream of mTORC1(Samtor)	—	—	up	7.05E-06
Q0KID5	CG34112-PA(CG34112)	—	—	up	2.59E-04
M9PGG8	CG17493	—	—	up	3.64E-03
Q9W022	CG8993-PA(CG8993)	—	—	up	4.44E-03
Q8SXD7	mitochondrial ribosomal protein S11(mRpS11)	small subunit ribosomal protein S11	Ribosome	down	4.97E-02
Q9VMQ6	CG31648-PA(CG31648)	cytochrome c oxidase assembly protein subunit 11	Oxidative phosphorylation	down	4.69E-02
Q9VBF0	CG5447-PA(CG5447)	—	—	down	4.54E-02
A4V4D2	tomosyn, isoform H(Tomosyn)	syntaxin-binding protein 5	—	down	2.68E-02
Q95NP8	Attacin A(AttA)	—	—	down	2.56E-02
Q9V535	CG8781	RNA-binding protein 8A	RNA transport	down	2.17E-02
			mRNA surveillance pathway		
			Spliceosome		
Q9VXB5	39S ribosomal protein L22, mitochondrial(mRpL22)	large subunit ribosomal protein L22	—	down	1.91E-02
O76926	KIN17 protein(kin17)	DNA/RNA-binding protein KIN17	—	down	1.42E-02
A0A0B4LHC3	Rho GTPase activating protein at 92B(RhoGAP92B)	—	—	down	7.62E-03
Q8T412	Malate dehydrogenase(CG10749)	malate dehydrogenase	Citrate cycle (TCA cycle)	down	6.79E-03
			Cysteine and methionine metabolism		
			Pyruvate metabolism		
			Glyoxylate and dicarboxylate metabolism		
			Carbon fixation in photosynthetic organisms		
			Carbon metabolism		
Q9VYN1	Protein kinase C delta(Pkcδ)	novel protein kinase C delta type	Chemokine signaling pathway	down	5.00E-03
			Vascular smooth muscle contraction		
			Tight junction		
			Fc gamma R-mediated phagocytosis		
			Neurotrophin signaling pathway		
			Inflammatory mediator regulation of TRP channels		
			GnRH signaling pathway		
			Estrogen signaling pathway		
			Type II diabetes mellitus		
			Insulin resistance		
			AGE-RAGE signaling pathway in diabetic complications		
Q58CL2	Secretory Pathway Calcium ATPase(SPoCk)	Ca2+-transporting ATPase	—	down	4.46E-03
Q8SX43	Sulfhydryl oxidase(Augmenter of liver regeneration,Alr)	mitochondrial FAD-linked sulfhydryl oxidase	—	down	1.92E-03
Q9V8I2	Ragulator complex protein LAMTOR2 homolog(CG5189)	ragulator complex protein LAMTOR2	mTOR signaling pathway	down	3.62E-05
Q8T4D6	Mitochondrial aconitase 2(mAcon2)	aconitate hydratase	Citrate cycle (TCA cycle)	down	9.66E-08
			Glyoxylate and dicarboxylate metabolism		
			Carbon fixation pathways in prokaryotes		
			Carbon metabolism		
			2-Oxocarboxylic acid metabolism		
			Biosynthesis of amino acids		
Q9VH66	CG8500	DIRAS family, GTP-binding Ras-like 2	—	down	9.84E-15

Among these proteins, Q9V8I2, Q9VH66, and Q9W138 are known to be related to mTOR pathway responding to nutritional signals and MAPK pathway. Q8T412 and Q8T4D6 are the enzymes in TAC involved in energy metabolism, and the changes in their expressions are consistent with the restriction of nutritional intake. Q9VYN1, Q8SX43 and Q9VMQ6 are involved in cellular antioxidant processes. It is worth noting that PKD interaction with Q9VYN1 has been reported to be related to male lifespan and starvation sensitivity. Q9VYN1, Q8SX43 and Q9VMQ6 are involved in cellular antioxidant processes. Q95NP8 protects against Gram-negative bacteria and exerts high oxygen resistance. Several DEPs having GTPase activity are also among these proteins. It is speculated that these DEPs may be involved in some kind of intracellular signal transfer. These common DEPs can be used as candidate DR regulators. However, a large portion of DEPs were in the day7 up- but day42 down-regulation region, day7 down- but day42 up-regulation region, indicating that DR may have different regulation on these proteins at different ages (Fig 2A). Some of these proteins are annotated to participate in the DR pathway. Such as Q9W590 had GTPase activity and regulated insulin receptor signaling pathway. Q7KNQ9 participated in MAPK signaling pathway. P92208 was related to JNK stress and involved in MAPK, IIS, Hippo pathway and apoptosis.

The majority of these above mentioned 20 proteins are known to be relevant to aging, such as in mTORC1 and Ras/MAPK signaling pathways, antioxidant, DNA damage repair, autophagy. It suggests that these proteins could play important roles during DR in maintaining health span and delaying the process of aging, and therefore these can be used as candidates for further research on DR.

### 3.8 Integrated analysis of transcriptome and proteome

**Fig 5:**
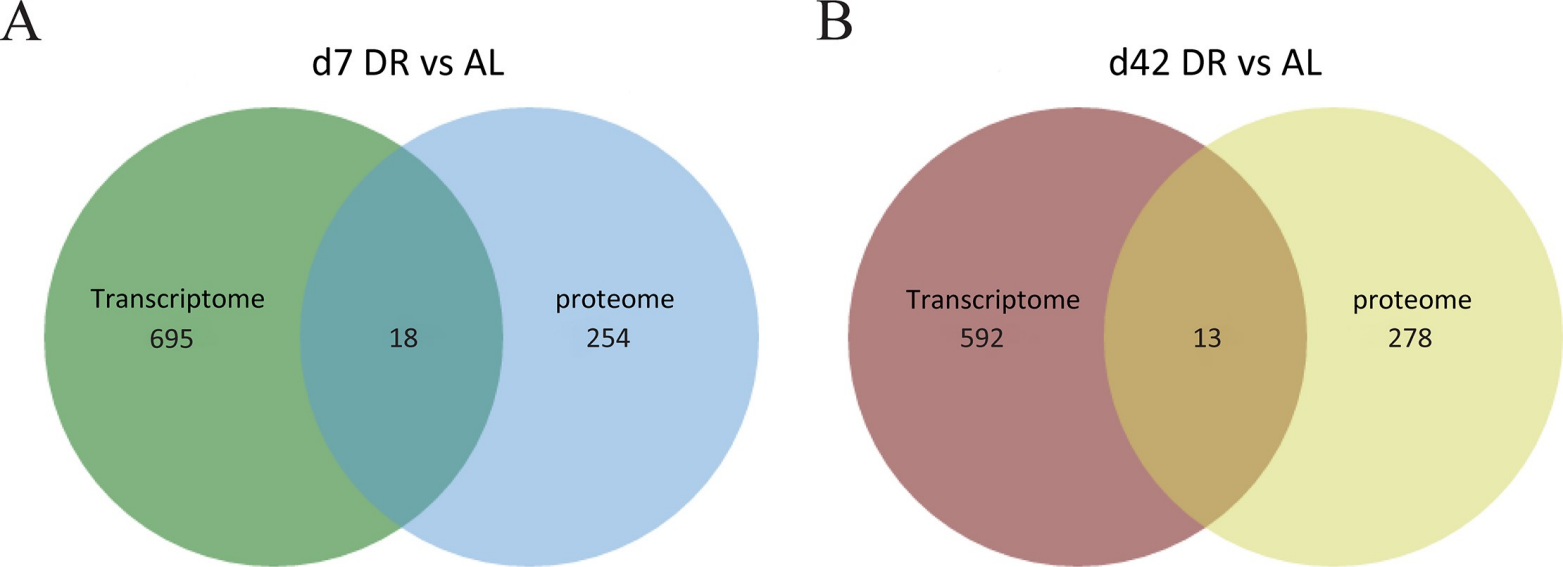
Differentially expressed genes comparison. DEGs were compared between transcriptional level and translational level with Venn diagrams. (A) day 7 DR vs AL, (B) day 42 DR vs AL.

In order to find out the common genes which are consistently regulated in fruit fly during DR at the transcriptional and translational levels, we conducted a combined analysis of proteome and transcriptome data that we reported previously at the same time point of d7 and d42 under DR condition [3]. We identified 18 common genes at day 7 DR vs AL, and 13 common genes at day 42 DR vs AL in both the transcriptome and the proteome (Fig 5A and 5B, S7 Table). Among them, a total of 15 genes were up- or down-regulated in both the transcriptome and the proteome (Table 3). It was worth noting that some of these proteins were related to longevity and age-related diseases. For example, Q8SYJ2 (ND-MLRQ) was NADH dehydrogenase MLRQ subunit participating in oxidative phosphorylation and its homologous gene Ndufa4 mutation promoted diet-induced diabetes [38]. Q8SYJ2 was also annotated to participate in NAFLD and neurodegenerative diseases. Q8MS59 (wat) had fatty-acyl-CoA reductase (alcohol-forming) activity and participates in peroxisome and together with O97125 (Hsp68) that was mentioned again participate in longevity regulating pathway according to KEGG annotations. It is speculated that these proteins that were stably regulated at the transcriptome and the proteome levels under DR may be important candidates for understanding the effect of DR.

**Table 3:** Common DEPs in both proteome and transcriptome at day 7 or day 42 between DR vs AL.

comparison	Tran_id	Prot_id	GO_fun	KEGG Annotations		Tran (log2fc)	Tran (pval)	Protein (log2fc)	Protein (pval)
				Definition	KEGG pathway				
d7 DRvsAL	FBgn0264776	Q8IND7	-	-	-	7.26E-01	6.00E-04	3.83E-01	3.44E-03
	FBgn0052230	Q8SYJ2	membrane	NADH dehydrogenase (ubiquinone) 1 alpha subcomplex subunit 4	Oxidative phosphorylation	5.13E-01	1.04E-02	2.88E-01	2.86E-02
					Non-alcoholic fatty liver disease (NAFLD)				
					Alzheimer's disease				
					Parkinson's disease				
					Huntington's disease				
	FBgn0013467	Q95T29	cell periphery	-	-	6.69E-01	3.13E-02	3.17E-01	1.58E-02
	FBgn0039620	Q8MS59	fatty-acyl-CoA reductase (alcohol-forming) activity	alcohol-forming fatty acyl-CoA reductase	Longevity regulating pathway—worm	6.15E-01	3.35E-03	2.85E-01	3.03E-02
					Peroxisome				
	FBgn0050035	A1Z8N1	intrinsic to membrane	facilitated trehalose transporter	-	8.99E-01	3.00E-03	2.82E-01	3.23E-02
	FBgn0001230	O97125	cellular developmental process	heat shock 70kDa protein 1/8	Spliceosome	1.17E+00	1.81E-02	2.71E-01	3.98E-02
					MAPK signaling pathway				
					Protein processing in endoplasmic reticulum				
					Endocytosis				
					Longevity regulating pathway—multiple species				
					Antigen processing and presentation				
					Estrogen signaling pathway				
					Legionellosis				
					Toxoplasmosis				
					Measles				
					Influenza A				
					Epstein-Barr virus infection				
	FBgn0037288	Q4V5T1	-	-	-	4.76E-01	2.86E-02	3.49E-01	7.93E-03
	FBgn0035665	Q9VRS7	-	-	-	9.78E-01	5.00E-05	2.86E-01	2.98E-02
	FBgn0031801	Q8SXE7	-	-	-	5.18E-01	2.84E-02	2.83E-01	3.20E-02
	FBgn0004921	P38040	membrane	-	-	8.11E-01	4.40E-02	3.68E-01	4.97E-03
	FBgn0033446	A1Z7Z4	-	-	-	1.09E+00	5.00E-05	2.66E-01	4.38E-02
d42 DR vs AL	FBgn0036364	Q9VU75	-	-	-	5.83E-01	2.01E-02	8.26E-01	1.40E-05
	FBgn0012042	Q95NP8	multi-organism process	-	-	-1.15E+00	2.00E-04	-7.63E-01	3.03E-04
	FBgn0037386	Q8T0T6	intrinsic to membrane	-	-	-9.05E-01	1.10E-03	-4.19E-01	4.88E-02
	FBgn0035542	A8JNK7	RNA processing	-	-	1.04E+00	6.75E-03	4.17E-01	2.73E-02

## 4. Discussion

In this study, LC-MS/MS was used to determine the proteome of *Drosophila* at day 7 and day 42 under DR and AL conditions. iTRAQ sequencing results were analyzed using Uniprot database. A total of 18629 unique peptides and 3662 proteins were identified, among which the DEPs accounted for about 10%. These DEPs are involved in hundreds of pathways, including DR classic pathways such as MAPK, insulin/PI3K/Akt, mTOR, FoxO, Wnt, P53, etc., which verifies the reliability of the sequencing results to a greater extent. We found that twenty common DEPs at both day 7 and day 42 may play an impotent role in DR. We also found 15 genes that were stably regulated by DR at both transcriptional and translational levels. These results provided useful data set for explaining the mechanism of DR and aging.

### DEPs constantly expressed during DR

Among 20 proteins, Q9VYN1(Protein kinase C δ, PKC-δ) was specifically involved in self-learning in *Drosophila* [39]. PKCδ interacted with Protein Kinase D (PKD) in *Drosophila*, which encoded a Ser/Thr kinase of the PKC family of Ca2^+^]/calmodulin-dependent protein kinases. Interestingly, PKD null alleles male, but not female fly, were slightly shorter lived and starvation sensitive [40]. In mammal, PKCδ activated PKD1 in response to phospholipase D activation signals on the mitochondrial membrane, and further inhibited mitochondrial depolarization and reduced the release of cytochrome C, thereby protecting cells from apoptosis and generally preventing oxidative damage [41]. Similarly, Q8SX43 is involved in oxidation-reduction process and tissue regeneration. Q9VMQ6 participated in Oxidative phosphorylation whereas O76926 participated in DNA replication and cellular response to DNA damage stimulus.

There are three common proteins involved in the mTORC1 signaling pathway. Q9V8I2 (LAMTOR2, also known as p14), which is always down-regulated under DR in our sequencing results, is a member of the trimeric p14, p18, and MP1 protein complex. It is reported that the trimeric protein complex interacts with Rag (The rag part of the Rag GTPases complex) and is essential for the activation of TORC1 by amino acids in mammalian and *Drosophila* cells [42]. Q9V8I2 is a positive regulator of Ras/MAPK and mTORC1 pathway [43] and is also essential for the formation of autolysosome in xenophagy (known as antibacterial autophagy) that resists the invasion of group A streptococci (GAS) and salmonella [44]. Another protein Q9VH66, had several human homologous genes, including DIRAS2 and DIRAS1, and had Ras GTPases activities. Interestingly, DIRAS2 and DIRAS1 induce and regulate autophagy by inhibiting Ras/MAPK and AKT1/mTOR signaling pathways and regulate the nuclear localization of the autophagy-related transcription factors FOXO3/FOXO3A and TFEB [45]. It is speculated that Q9VH66 with Ras GTPases activities also plays an important role in metabolism and longevity in DR. It is worth noting that Q9W138 (SAMTOR), which is consistently up-regulated at d7 and d42 DR vs AL, negatively regulates mTORC1 signaling [46]. In cells, S-adenosylmethionine (SAM) can directly binds to SAMTOR. However, when methionine was starved, SAM level was reduced. It results in the promotion of the combination of SAMTOR and GATOR1, thereby inhibiting mTORC1 signaling in a dose-dependent manner [45]. Due to the reduction of total amino acid intake under DR, it is speculated that the benefit of life extension from DR may be partly dependent on SAMTOR-mediated inhibition of mTORC1.

Q95NP8 (Attacin-A) encodes an antibacterial peptide with activity against Gram-negative bacteria [47]. Up-regulation of Attacin conferred tolerance to severe hyperoxia [48]. Previous studies showed that increased oxidative stress occurred during aging, age-related neurodegenerative diseases and cancer [49,50]. In our study, however, DR down-regulates the expression of Attacin-A in all ages. It will be interesting to explore the relationship between DR and the sensitivity and tolerance of organisms to oxides and the antioxidant effect of organisms.

A4V4D2 had GTPase activator activity and Rab GTPase binding activity. Similarly, A0A0B4LHC3 had GTPase activator activity and Rac GTPase binding activity. Q9VH66 had GTPase activity and is involved in small GTPase mediated signal transduction. Q58CL2 had calcium transmembrane transporter activity and proton-exporting ATPase activity. We proposed that these uncharacterized proteins may be involved in important intracellular signal transduction. Q8T412 (Malate dehydrogenase), and Q8T4D6 (Mitochondrial aconitase 2), both participates in tricarboxylic acid cycle (TAC) and it points towards changes in energy metabolism caused by DR.

Our results suggest that these proteins could play important roles during DR in maintaining health and delaying aging, and can therefore be used as candidates for further research on DR.

### Spliceosome is linked to age-related disease and DR

The spliceosome is a kind of multi-component complex around 60S in size, mainly composed of small nuclearRNA (snRNA) and protein. It is interesting that both spliceosome and snRNA appeared in our annotations and enrichment results at a very high level at both day 7 and day 42. Spliceosomes are formed during the splicing process involving snRNA recognition and base pairing. Under the catalysis of RNA polymerase, DNA is transcribed into precursor messenger RNA (pre-mRNA), which contains introns. Subsequently, after removing introns and connecting exons by the action of spliceosomes in splicing step, pre-mRNA can be converted into mature mRNA. There are 5 ways of splicing, intron retention, variable 5 'splice site, variable 3' splice site, exon box, and mutually exclusive exons [51]. The diversity of splicing methods and the complexity of pre-mRNA have led to certain proteins that may have multiple subtypes, which is determined by the splicing process. The research hotspots on spliceosomes mainly focus on diseases. The mainstream view believes that many diseases are related to errors in splicing, such as Alzheimer's disease [31,32]. Inhibiting the spliceosome function in cancer cells can effectively suppress malignant cancers driven by the MYC [30]. Similarly, RNA splicing homeostasis also played an important role in DR longevity. Splicing factor 1 exerts the longevity benefits of DR by regulating TORC1 pathway components AMPK, RAGA-1 and RSKS-1/S6 kinase in *C*. *elegans* [28]. Since RNA splicing is a fundamental step connecting transcription and translation, the homeostasis of this process is critical to the normal physiological function in aging or age-related diseases. Although there are some clues that spliceosome plays an important role in DR, current research linking DR and spliceosome is still in its early stages. According to KEGG pathway analysis, we have summarized the DEPs involved in the spliceosome during DR, so as to further study the role of the spliceosome in DR.

### Neurodegenerative diseases regulated by DR

Both d7 and d42 DR vs AL KEGG annotation results show that multiple DEPs are involved in the pathways associated with the three classic neurodegenerative diseases which are Alzheimer's disease, Parkinson's disease, and Huntington's disease. Previous studies have observed that the symptoms of Alzheimer's disease and Parkinson's disease are alleviated after DR [52]. Drugs that simulate DR, such as resveratrol, have been found to be effective in alleviating symptoms of these three neurodegenerative diseases and improve cognitive ability [53]. However, no research has clearly pointed out the mechanism or key factors so far. In this study, DEPs annotated in three classic neurodegenerative diseases at d7 and d42 DR vs AL may be critical proteins for the regulation of neurodegenerative diseases during DR.

### DR regulates the ubiquitination process

Previous studies have shown that DR exerts its benefits through epigenetic modification. For examples, DR is generally strongly protective against age-related changes in DNA methylation in mice [54] and DR is related to histone acetylation regulated by sirtuin [55]. The ubiquitination pathway has been reported to participate in longevity determination in response to DR [12]. In this study, our results also suggested that ubiquitination may play an important role during DR. Ubiquitin is composed of 76 amino acids and is highly conserved. Ubiquitination is the ubiquitous way of endogenous protein degradation to maintain the intracellular homeostasis in eukaryotic cells [56]. Note that the results of both GO enrichment and KEGG pathway annotation showed that relatively large number of DEPs participate in ubiquitination at d42 DR vs AL. Among them, Q8MQN4 encodes a HECT-type E3 ubiquity ligase and is involved in immune response against Gram-positive bacteria mediated by Toll pathway [57]. Q8MQN4 homologous gene HERC3 in humans participates in the regulation of cell senescence through ΔNp63α/HERC3/MM1/c-Myc axis [58]. Multiple DEPs participate in the ubiquitination pathway suggesting that ubiquitination may play a more profound role under DR.

### Autophagy related pathways were annotated during DR

Autophagy is the process by which cells digest part of themselves through lysosomes and autophagosomes to maintain intracellular homeostasis. Under normal circumstances, autophagy occurs at low basal levels [59] and it is upregulated by inducing factors, which can be divided into extracellular factors such as hypoxia, growth factor withdrawal, etc. and intracellular factors including the damaged organelles, misfolded proteins, ER stress, etc. [60]. Studies on cells have shown that starvation [61], DNA damage [62], and radioactive elements [63] can all induce autophagy. DR also induces autophagy and it is essential for extending lifespan [64]. At present, pathways related to autophagy are PI3K/Akt, EcR and mTOR [65,66]. In this study, many DEPs were not only found in the PI3K/Akt and mTOR pathways, but also involved in lysosomes according to the GO enrichments and KEGG pathway annotations, which provided clues for further research on the mechanism of DR induced autophagy.

### Riboflavin metabolism is regulated in old age under DR

Riboflavin metabolism was the most enriched KEGG pathway in d42 DR vs AL group. Riboflavin is a component of prosthetic group of flavin enzymes in the organism, and it provides hydrogen ions in the redox reaction [67]. Riboflavin mainly participates in physiological processes including respiratory chain, lipid oxidation, synthesis of protein and certain hormones, etc. In addition, it can also promote development and cell regeneration [68,69]. At present, there is no research on riboflavin related to DR. In this study, all DEPs in riboflavin metabolism were up regulated by DR at day 42. It speculated that oxidation reactions involving flavin-dependent oxidases may be more active in old flies under DR.

We also discovered that many DEPs are involved in the longevity and age-related disease pathways. It suggests that these proteins may be involved in lifespan determination and delaying the occurrence and deterioration of age-related diseases. This calls for further verification on the DR. In the integrated analysis of transcriptome and proteome, we found that some proteins were stably regulated at the transcriptional and translational levels under DR. However, similar to previous reports [70,71], we also found that the correlation between transcriptome and proteome was very low. Besides, only a small proportion of genes overlapped between the proteomics (DEPs) and transcriptomics (DEGs) analysis. Interestingly, some of common genes were up-regulated in the proteomics analysis while down-regulated in the transcriptomic analysis, or vice versa. It implies that there may be some post-transcriptional regulatory mechanisms that regulate the final expression of these genes into proteins. The inconsistency in transcript and protein levels could be partially explained by the reason that the buffer of mRNA fluctuation, transcript levels were not sufficient to predict protein levels [70]. In addition, post-transcriptional modifications often cause differences in expression at the RNA and protein levels [70].

The iTRAQ technology used in this study has some advantages and disadvantages. The iTRAQ quantitation method could cause underestimation of protein ratios. In contrast, the label-free method was more accurate than the iTRAQ method [22]. However, the two biological replicates designed in this study can match 8-plex iTRAQ, which conducts a one-time proteomics experiment on a single run to analyze 8 samples. This can reduce technical errors. In this study, two time points were measured giving an insight of the effect of DR on fruit fly in the young and old stages. This study also provided a useful data set for further investigation on the mechanism of aging and DR.

## Supporting information

S1 Fig Volcano plots showing all proteins change. (DOCX)

S1 Table Concentration of protein samples. (XLSX)

S2 Table Lifespan data for Fig 1A. (XLSX)

S3 Table Peptide identification list. (XLSX)

S4 Table Protein identification list. (XLSX)

S5 Table Protein quantification and differential analysis. (XLSX)

S6 Table Genes and pathways analysis. (XLSX)

S7 Table Integrated analysis of transcriptome and proteome. (XLSX)
