# Supplementary material for: Comparative proteomics analysis of dietary restriction in Drosophila
Source: PLoS One. 2020 Oct 16;15(10):e0240596. doi: 10.1371/journal.pone.0240596 (PMC7567386; doi:10.1371/journal.pone.0240596)

**Comparative Proteomics** [**Analysis**](https://xueshu.baidu.com/usercenter/paper/show?paperid=ad93aa557f52fbf6cf6d4fd313a4ad85&site=xueshu_se) **of** **Dietary Restriction in *Drosophila***

Gao Yue^†^, Chenxing Zhu^†^, Li Keqin, Xingyi Cheng, Yanjiao Du, Yang Deying, Fan Xiaolan, Uma Gaur, Mingyao Yang *

Supplementary Figure

Figure S1. Volcano plots showing all proteins change. The DR treatment at day 7 (A) and day 42 (B). Proteins changed with age under AL (C) and DR (D) conditions. Horizontal dashed line indicates a significance cutoff of p< 0.05 and vertical dashed lines indicate fold change >1.2 and <0.8 (n=2).


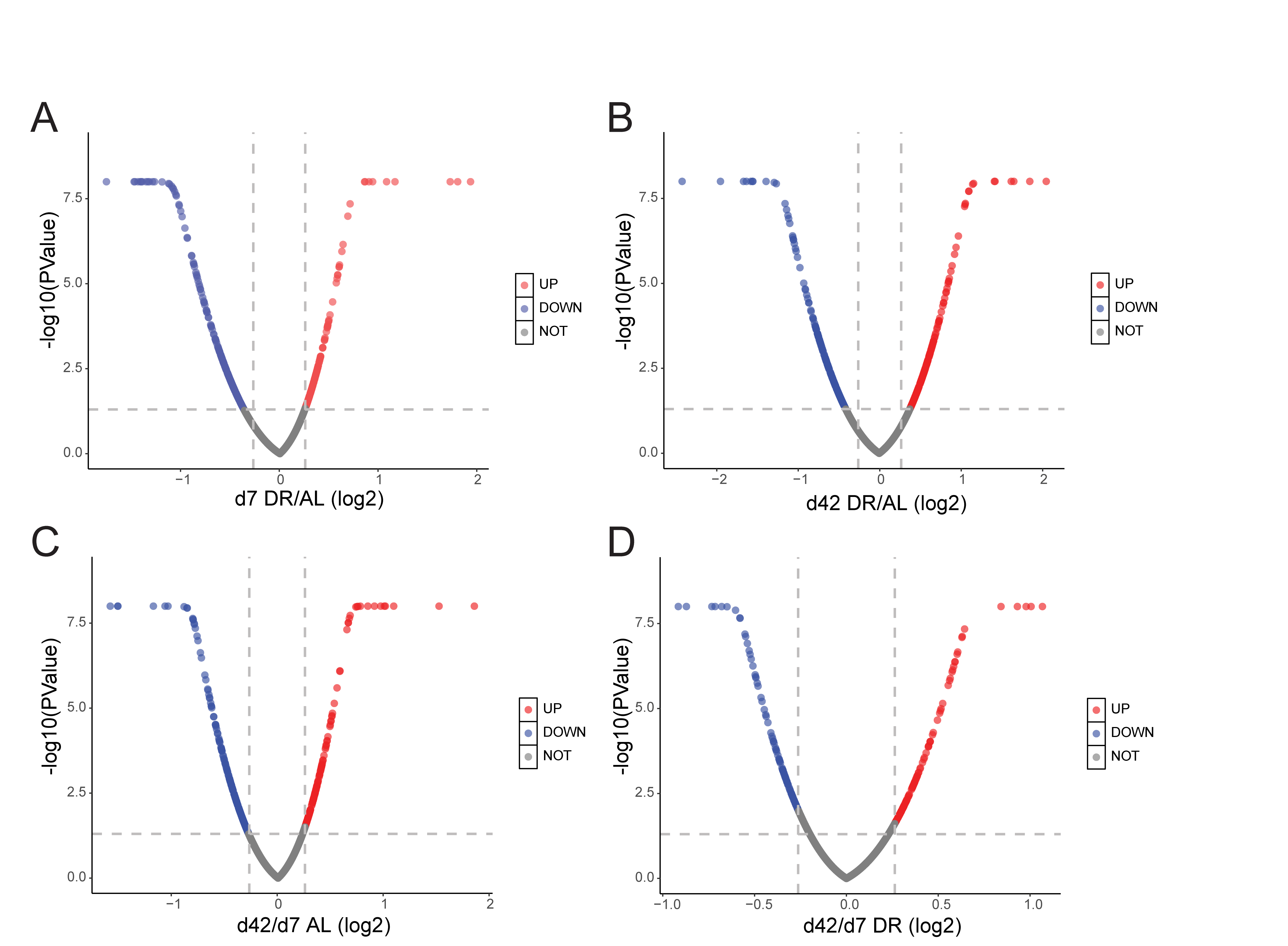

Supplement: S1 Fig — (DOCX) [file pone.0240596.s001.docx]
